# Kub5-Hera, the human *Rtt103* homolog, plays dual functional roles in transcription termination and DNA repair

**DOI:** 10.1093/nar/gku160

**Published:** 2014-03-03

**Authors:** Julio C. Morales, Patricia Richard, Amy Rommel, Farjana J. Fattah, Edward A. Motea, Praveen L. Patidar, Ling Xiao, Konstantin Leskov, Shwu-Yuan Wu, Walter N. Hittelman, Cheng-Ming Chiang, James L. Manley, David A. Boothman

**Affiliations:** ^1^Simmons Cancer Center, University of Texas Southwestern Medical Center, Dallas, TX 75390-8807, USA, ^2^Department of Biological Sciences, Columbia University, New York, NY 10027, USA, ^3^Laboratory of Genetics, Salk Institute of Biological Studies, La Jolla, CA 92037, USA, ^4^Department of Radiation Oncology, Case Western Reserve University, Cleveland, OH 44106, USA and ^5^Department of Experimental Therapeutics, M.D. Anderson Cancer Center, Houston, TX 77030, USA

## Abstract

Functions of Kub5-Hera (In Greek Mythology Hera controlled Artemis) (K-H), the human homolog of the yeast transcription termination factor Rtt103, remain undefined. Here, we show that K-H has functions in both transcription termination and DNA double-strand break (DSB) repair. K-H forms distinct protein complexes with factors that repair DSBs (e.g. Ku70, Ku86, Artemis) and terminate transcription (e.g. RNA polymerase II). K-H loss resulted in increased basal R-loop levels, DSBs, activated DNA-damage responses and enhanced genomic instability. Significantly lowered Artemis protein levels were detected in K-H knockdown cells, which were restored with specific K-H cDNA re-expression. K-H deficient cells were hypersensitive to cytotoxic agents that induce DSBs, unable to reseal complex DSB ends, and showed significantly delayed γ-H2AX and 53BP1 repair-related foci regression. Artemis re-expression in K-H-deficient cells restored DNA-repair function and resistance to DSB-inducing agents. However, R loops persisted consistent with dual roles of K-H in transcription termination and DSB repair.

## INTRODUCTION

Maintaining genomic stability through progressive cell-cycle divisions is essential for survival. Genomic instability can arise by several different mechanisms (1), ultimately leading to mutations and/or chromosomal rearrangements that contribute to disease states, such as cancer (2). One unrepaired DNA double-strand break (DSB) can cause lethality (3). Mis-repaired DSBs are also a prominent source of chromosomal rearrangements, resulting in translocations within the genome (4). Thus, DSBs are a constant threat to genomic stability and can arise naturally during normal metabolic, replication and/or developmental processes (5). Another prominent, yet at this point understudied, mechanism for genomic instability is through the formation of persistent RNA:DNA hybrids, known as R loops (6). R loops are an evolutionarily conserved consequence of transcription that form under a variety of conditions, and if not properly resolved lead to DSBs and genetic instability (6,7). In transient forms, R-loop formation is an essential process in numerous normal cellular processes, such as class switch recombination (8), and may also contributes to normal transcription termination by RNA Polymerase II (RNAPII) (9–11).

Transcription termination by RNAPII is a complex process requiring multiple protein factors (12). Interestingly, termination factors are linked to several different disease states. Senataxin, a putative DNA:RNA helicase, is highly associated with neurological pathologies, such as amyotrophic lateral sclerosis 4 and ataxia with oculomotor apraxia 2 (13,14). Polymorphisms in Xrn2, a 5′–3′ exoribonuclease, are associated with cases of spontaneous lung cancer in non-smokers (15). PSF, together with p54(nrb), functions in the recruitment of Xrn2 (16) and is critical for cellular survival in colon and prostate cancers (17,18). p54(nrb) is highly expressed and required for development and progression of malignant melanoma (19).

Along with roles in transcription termination, PSF, p54(nrb) and Senataxin are also implicated in the DNA-damage response (DDR), particularly in response to DSBs. PSF and p54(nrb) have direct functional roles in both the non-homologous end-joining (NHEJ) and homologous recombination (HR) pathways of DSB repair (20–22). Loss of either PSF or p54(nrb) abrogates DNA-repair kinetics and leads to increased chromosomal aberrations (21,23). Senataxin is also implicated in the DDR, and in particular, in response to DSBs created by R loops formed during various stages of transcriptional pausing (24–26).

In light of the complex nature of the RNAPII termination process, it is likely that factors in addition to those mentioned above are involved. In yeast cells, an additional factor, Rtt103 contributes to transcription termination (27). Rtt103 interacts with both RNAPII, via the C-terminal domain (CTD) of its largest subunit, and Rat1, the yeast homolog of Xrn2. Rtt103 facilitates the exonuclease activity of Rat1 in termination (27). The human homolog of Rtt103 is Ku70 binding protein #5-Hera (K-H), also known as RPRD1B or CREPT (28,29). Similar to Rtt103, K-H interacts with RNAPII (29). K-H also mediates expression of Cyclin D1 and RNAPII occupancy at the 3′-end of genes (28). Rtt103 has also been implicated in the DDR, yet its functions and involvement in specific DNA-repair pathways remain unknown (30). While K-H overexpression may also promote cell proliferation and tumorigenesis (28), these functions within the cell also remain undefined.

Here, we employ genetic and biochemical techniques to uncover novel functions of K-H. We report that K-H functions in RNAPII regulation, and aids in stabilizing interactions between transcription termination factors, localizing Xrn2 to the 3′-end of genes and ultimately suppressing R-loop formation. Importantly, we report that K-H forms additional complexes and has additional and separate functions in DSB repair through stabilization of the DNA-repair factor, Artemis.

## MATERIALS AND METHODS

### Cell culture

shScr, shk-h and all variations of cells were grown in DMEM with 15% FBS, L-glutamine, 100 µg/ml hygromycin and 1 µg/ml puromycin in a 10% CO_2_–90% O_2_ air humidified atmosphere at 37°C. shScr and shk-h knockdown cells were grown as described above, except under selection with 1 µg/ml puromycin. shScr and shk-h-MDA231 and all variations of cells were grown in DMEM with 5% FBS and L-glutamine in a 10% CO_2_–90% O_2_ air humidified atmosphere at 37°C.

### Mouse embryonic fibroblast production

K-H heterozygote males and females were used for timed mating. At 12.5 p.c., a female mouse was sacrificed and embryos removed. Individual embryos were then minced and treated with trypsin at 37°C for 1 h to separate cells. The cell slurry was then plated into a T75 tissue culture flask and allowed to attach and grow. These cells were maintained in DMEM with 5% FBS and at 10% CO_2_.

### Antibodies used

An antibody recognizing H2AX (α-total H2AX, BL179) and 53BP1 (A300-272 A) was purchased from Bethyl Laboratories (Montgomery, TX). γ-H2AX phospho-specific antibody (JBW301) was obtained from Millipore (Billerica, MA). α-Artemis antibodies (E-18, K-14), and actin (C-11) were obtained from Santa Cruz Biotech (Santa Cruz, CA). α-Ku70 and α-Ku70/80 heterodimer specific antibodies (N3H10 and 162, respectively) were purchased from Genetex (Irvine, CA). K-H monoclonal antibody was produced at Case Western Reserve University. K-H polyclonal antibody (SAB1102247) was purchased from Sigma. S9.6, an antibody specific for Rloops (RNA:DNA hybrids) was provided by Dr Stephen H. Leppla (NIH, Bethesda, MD). PSF (A301-320A) and Xrn2 (A301-103A) are from Bethyl Laboratories (Montgomery, TX). RNAPII antibodies (sc-56767) is from Santa Cruz Biotech (Santa Cruz, CA). 

## Colony-forming assays

shScr, shScr-MDA231, shk-h and shk-h-MDA231 and all variations of these cells were plated onto 60-mm tissue-culture plates and allowed to grow for 2 days. Cells were then exposed to IR (at various doses as indicated), allowed to grow for 7 days, washed with PBS and stained with crystal violet solution. Colonies with >50 normal appearing cells were counted and percent survival calculated and graphed with dose.

### Neutral comet assay

Assays were performed as per manufacturer’s instructions (Trevigen) (Catalog# 4250-050-K). Briefly, cells were mock or IR treated and allowed to recover for indicated times. After harvesting, cells were mixed with low melting agarose. Cells were then exposed to electrophoresis and stained with SYBR®-green to detect released DNA. Cells were then imaged and comet tail lengths measured by NIH Image J.

### Immunofluorescence

In order to visualize 53BP1, γ-H2AX and Artemis, cells were plated and grown to 70% confluence on glass coverslips and either mock- or IR-treated. Cells were then washed once with PBS, permeabilized and fixed in methanol/acetone (70/30, v/v). Cells were then blocked in PBS containing 5% FBS for 30 min at room temperature. Cells were then washed three times with PBS and exposed to primary antibody for 1 h at room temperature as indicated. Cells were washed three times with PBS, exposed to secondary antibody for 30 min at room temperature, washed three times with PBS and mounted onto glass slides. Detection of R loops using the S9.6 antibody was performed as previously described (31). Visualization was performed using a 100X oil objective with fluorescence on a Nikon microscope.

### Plasmid re-ligation assays

The pYes2 plasmid DNA from Invitrogen was digested with the following restriction enzymes: BamHI, SacI, PvuII and BamHI + SacI at 37°C for 45 min. Cut and uncut DNAs were purified from Agarose gels using Qiagen Extraction kits. Linearized and control circular plasmid DNAs were then used in PEG/LiAc-based yeast transformations from Clontech (Yeastmaker Yeast Transformation System 2). Overnight YPDA cultures (25 ml) were grown to an OD_600_ of 0.25, harvested and re-suspended in 50 ml YPDA and incubated for 4 h at 30°C until OD_600_ of 0.4–0.5 were reached. Competent yeast cells were generated as described (Clontech) and transformed with equivalent amounts of uncut or restriction enzyme-digested pYes2 plasmid DNA as indicated. Transformation reactions were performed using plasmid DNA concentrations of 10 µg in 50 µl competent *rtt103Δ*, *hdf1Δ*, wild-type (WT) and *rtt103Δ* yeast stably corrected with yeast RTT103 and human K-H cDNA (hK-H). Briefly, after 42°C heat shock to promote DNA transformation, yeast were harvested and resuspended in 0.5 ml 0.9% NaCl and plated onto SD–URA selection plates. Colonies were scored 3 days later and values graphed as means*±*standard errors for three separate transformation experiments.

### Mammalian plasmid re-ligation assays

The pEGFP-Pem1 plasmid was digested with HindIII or I-SceI for 8–12 h to generate free DNA ends. pCherry plasmid was co-transfected with linearized DNA to control for transfection efficiency. shScr and shk-h cells were transfected at roughly 20–25% confluency and allowed to grow for 3 days. Transfections were performed using Lipofectamine-2000 using manufacturer’s instructions. Flow cytometry was performed using a Beckman–Coulter Cytomic FC 500.

### Metaphase spreads and chromosome aberration analyses

Exponentially growing cells were mock- or IR-treated. Cells were returned to 37°C for 30 min to allow for mitotic exit (particularly IR-exposed cells) and then incubated with colcemid (1 µg/ml) for 2 h for mitotic cell selection before harvest. Harvested cells were fixed in hypotonic solution containing 75 mM KCl and fixed in methanol:acetic acid (1:1 v/v). Metaphase spreads were prepared, stained with Giemsa and examined by light microscopy. Metaphase spreads (>50) were then scored for chromosome breaks, gaps and aberrations.

### HeLa whole-cell extract preparation and gel-filtration chromatography

**Figure 1.**
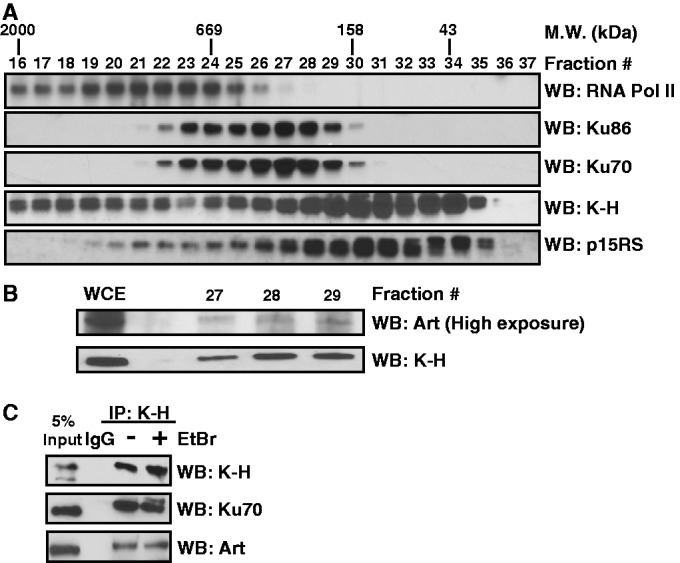
K-H forms a complex with NHEJ factors. (A and B) To interrogate potential *in vivo* K-H interacting partners exponentially growing HeLa cells were collected and lysed for FPLC. Individual fractions were separated by SDS-PAGE and western blot techniques were used to probe for indicated proteins. (C) To validate K-H and NHEJ complex member interactions nuclear extracts from shScr cells were used to perform co-immunoprecipitation (co-IPs) experiments with and without EtBr (5 μM), to check for the contribution of DNA to these interactions. Co-IPs were separated by SDS-PAGE followed by western blot analysis for indicated NHEJ factor.

HeLa cells were cultured in two 150 mm^2^ dishes (up to ∼80% confluency) in DMEM supplemented with 5% FBS and 1 mM L-glutamine in a 5% CO_2_ humidified atmosphere at 37°C. Cells were trypsinized, harvested by centrifugation and washed with cold 1X PBS. Cells were resuspended in 1 ml extraction buffer [25 mM Tris–HCl (pH 7.7), 2 mM MgCl_2_, 100 mM NaCl, 10 mM β-glycerophosphate, 5 mM NaF, 0.5 mM Na_3_VO_4_, 10% glycerol, 0.1% NP-40, 1X protease inhibitor cocktail (Sigma), 100 units of turbonuclease (Fisher) and 1 mM DTT]. The cell suspension was incubated on ice for 5 min and passed through 1-ml syringe with 27 G needle until homogeneous suspension was obtained. The suspension was incubated on ice for 30 min followed by 10 min at 37°C. The cell lysate was centrifuged at 14 000 rpm for 30 min at 4°C using a microfuge. The supernatant was carefully collected as whole cell lysateand used for gel-filtration chromatography. Chromatography steps were carried out using AKTA Purifier 10 (GE Healthcare). For the fractionation of whole cell lysate, ∼3.0 mg of protein was loaded onto a 24-ml Superose 6 HR 10/30 column (GE Healthcare) pre-equilibrated with chromatography buffer [25 mM Tris–HCl (pH 7.7), 100 mM NaCl, 5% glycerol and 1 mM DTT] and run in the same buffer at a flow rate of 0.5 ml/min. Molecular weight standards (Pharmacia Biotech) were used to calibrate the column (as indicated in Figure 1A).

### Nuclear extract preparation

Cell pellets were re-suspended in Buffer A [10 mM Hepes (pH 7.9), 10 mM KCl, 0.1 mM EDTA (pH 8.0), 0.1 mM EGTA, 1.0 mM DTT, 0.5 mM PMSF] and allowed to swell for 10 min, 4°C. NP-40 was then added to cell solutions to a final concentration of 0.5% and vortexed at low intensity for 30 sec. Isolated nuclei were then harvested by centrifugation (2000× *g*), the nuclear pellets were resuspended in Buffer C [20 mM Hepes (pH 7.9), 0.4 M NaCl, 1.0 mM EDTA, 1.0 mM EGTA, 1.0 mM DTT, 0.5 mM PMSF] for 15 min at 4°C. Nuclear extracts were then isolated by centrifugation (25 000× *g*) for 15 min, and assessed for protein concentrations by Bradford assays.

### Immunoprecipitation and Chromatin Immunoprecipitation

Of nuclear extract, 0.5 to 1 µg was incubated with 5 µg of specified primary antibody conjugated to Protein A/G beads. Each experiment was washed three times with NETN solution [20 mM Tris–HCL (pH 8.0), 0.1 M NaCl, 1 mM EDTA, 0.05% NP-40]. After washes each sample was separated on 8% SDS-polyacrylamide gel. Immunoprecipitation and Chromatin Immunoprecipitation (ChIP) experiments were performed using previously described methods (16).

### Statistics

All experiments were performed three or more times in triplicate. Western and immunofluorescence images shown, are representative of these findings. Means and standard errors were calculated and differences between treatments were determined by confidence limit calculations using student’s *t*-tests. *P*-values (0.01 and 0.05) for 99% and 95% confidence limits were considered significant.

## RESULTS

### K-H biochemically interacts with NHEJ factors

Ku70 binding protein #5-Hera (K-H) was isolated in a screen intended to identify Ku70-binding partners involved in NHEJ using a similar yeast two-hybrid strategy as described (32). The association of K-H with Ku70 in these assays was relatively strong, comparable to the association of p53 with SV40 large T as measured by growth on selection media and beta-galactosidase activity (Supplementary Table S1). Deletion analyses indicated that a coiled-coil domain in K-H interacted with a comparable coiled-coil domain within Ku70, also known to associate with nuclear clusterin (33). K-H has two highly conserved functional domains: an amino-terminal CTD-interacting domain (CID), that mediates interactions with the CTD of RNAPII (27,29) and a carboxy-terminal coiled-coil domain (Supplementary Figure S1A), which is required for Ku70 interaction. A specific point mutation, L276A, within the coiled-coil domain of K-H abolished its binding to Ku70 (Supplementary Table S1). Human (hK-H) and mouse (mK-H) K-H share significant regions of homology with yeast Rtt103 as noted by Clustal Omega analyses (Supplementary Figure 1A).

Due to its association with Ku70, we used a plasmid-based DSB-repair assay to define possible repair deficiencies in *Rtt103Δ* yeast. Plasmids conferring growth on Uracil-deficient medium (*UraA* cDNAs) were digested with BamHI (5′-overhangs), SacI (3′-overhangs), a combination of BamHI and SacI, (BamHI/SacI, yielding incompatible 5′- and 3′-end overhangs) or PvuII (blunt ends) (Supplementary Figure S1B). Unlike yeast deficient in *Hdf1* (yKu70, the canonical yeast NHEJ protein), that were unable to repair any type of introduced DSB, *Rtt103Δ* yeast lacked the specific ability to repair DSBs with incompatible ends (i.e., complex or blunt DNA ends), which require additional DNA end-processing (Supplementary Figure S1B). Unlike prior reports stating that Rtt103 is not required for the end-joining process (30), our data strongly suggest that Rtt103 deficient cells exhibit a specific defect in the repair of incompatible or blunt DSB ends. Importantly, re-expression of either yeast Rtt103 or human K-H (hK-H) cDNAs restored the DNA repair capabilities of *Rtt103Δ* yeast (Supplementary Figure S1B). Interestingly, the DNA-repair defect noted in *Rtt103Δ* yeast closely resembled the DNA-repair deficiency of yeast lacking *Exo1*, a 5′–3′ exonuclease that is required for DNA end-processing during NHEJ (34), a similar but non-overlapping function performed by the human Artemis protein (35).

To confirm and explore additional K-H-binding partners, we performed gel filtration on HeLa whole cell extracts. Interestingly, two separate complexes containing K-H were readily detectable, suggesting relatively separate associations with RNAPII (fractions 16–22) (Figure 1A) and another complex closely associated with p15RS (fractions 27–35) (Figure 1A); an association between K-H and p15RS was previously reported (29). Additionally, K-H co-eluted with several known NHEJ factors, including Ku70, Ku86 and Artemis (fractions 27–29) (Figure 1A and B), consistent with yeast two-hybrid data suggesting an association of K-H with Ku70 (Supplementary Table S1). To visualize Artemis, fractions 27–29 were concentrated and probed for levels of K-H and Artemis by western blot analyses (Figure 1B). Importantly, co-immunoprecipitation (co-IP) analyses using a K-H-specific antibody generated in our laboratory, which recognizes K-H but, not the closely related p15RS protein (Supplementary Figure S2A), revealed that K-H associated with Ku70 and Artemis. Furthermore, this association was not due to interactions with DNA, since co-IPs were noted in the presence or absence of ethidium bromide (5 μM) (Figure 1C). DNase treatments also did not affect the co-IPs (not shown). These data confirm the initial yeast two-hybrid observation that K-H interacted with Ku70, and show that Ku86 and Artemis are present in higher molecular weight protein complexes with K-H, separate from RNAPII.

### Increased genomic instability and basal DSB formation due to K-H loss

**Figure 2.**
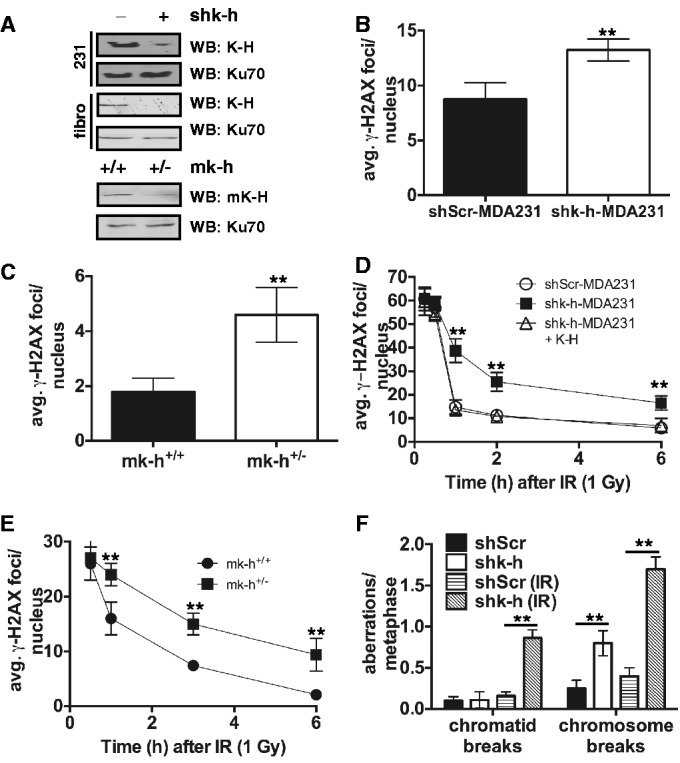
Loss of K-H leads to increased DSBs and genomic instability. (A) shScr, shk-h, shScr-MDA231, shk-h-MDA231, *mk-h*^+/+^ and *mk-h*^+/–^ cells were used to interrogate the degree of K-H loss each cell system. (B and C) Basal levels of the DNA damage indicator γ-H2AX were measured in shScr-MDA231, shk-h-MDA231, *mk-h*^+/+^ and *mk-h*^+/–^ cells by immunofluorescence (IF). (D and E) Rates of γ-H2AX disappearance were measured in shScr-MDA231, shk-h-MDA231, shk-h-MDA231 + K-H, *mk-h*^+/+^ and *mk-h*^+/–^ cells at indicated times after ionizing radiation (IR) exposure by IF, the first time point is 0.5 h after IR exposure. (F) Amounts of genomic aberrations were quantitated in shScr and shk-h cells with and without exposure to IR (2 Gy). (**P < 0.01).

To examine possible functions of K-H in DNA repair, we generated stable shRNA-K-H knockdown cell lines using human foreskin fibroblast (shk-h) and triple negative, metastatic MDA-MB-231 breast cancer (shk-h-MDA231) cells. Non-targeted shRNA-Scr fibroblast (shScr) and MDA-MB-231 (shScr-MDA231) control cells were generated at the same time. K-H-targeted shRNA knockdown was directed to a 5′-untranslated region (UTR) sequence to facilitate reconstitution of cells with human K-H cDNA. We also generated mouse embryonic fibroblasts (MEFs) from K-H wild type (*mk-h*^+/+^) or heterozygote (het) mice (*mk-h*^+/^^−^). We confirmed stable loss of K-H in knockdown or *mk-h*^+/^^−^ het cells, and showed that levels of Ku70 remained unchanged (Figure 2A). We used *mk-h*^+/^^−^ MEFs in our studies since complete loss of K-H led to early embryonic lethality (data not shown) and *mk-h*^+/^^−^ cells are haplo-insufficient for phenotypes examined below. In all three cell systems, loss of K-H led to increased basal levels of γ-H2AX and 53BP1 foci formed without genomic insult (Figure 2B and C and Supplementary Figure S2B). Furthermore, K-H deficient cells showed delayed disappearance of DDR biomarkers following exposure to IR (Figure 2D and E and Supplementary Figure 2C). The delay in DDR foci regression was confirmed by a delay in neutral comet tail regression after IR treatment in shk-h-MDA231 cells compared to shScr-MDA231 or K-H reconstituted shk-h-MDA231 cells (Supplementary Figure 2D). As neutral comet analysis examines DSBs specifically, we concluded that loss of K-H impaired the ability of affected cells to repair DSB lesions. We also observed significant increases in chromosomal aberrations, by metaphase spreads, before and after exposure to IR (Figure 2F and Supplementary Figure 3A) in shk-h knockdown fibroblasts compared to shScr cells. Elevated levels of chromatid aberrations were also noted in irradiated shk-h fibroblasts compared to shScr control cells (Figure 2F). In contrast, levels of other genomic aberrations, such as di-centric, tri- and tetra-radial chromosomes, were not statistically different between shScr and shk-h fibroblast cells (Supplementary Table S2). Collectively, these data suggested that K-H plays a role in mediating specific DSB-repair processes. However, direct or indirect role(s) for K-H in HR repair of DSBs in mammalian cells could not be ruled out, since increases in chromatid and chromosome breaks after IR were observed with loss of either HR or NHEJ DSB-repair pathways (36). Yet, the lack of radial chromosomes, commonly seen in cells that have lost the ability to perform HR; thus reverting to NHEJ for DSB repair (36,37), in shk-h cells suggest that the primary DSB repair defect in K-H-lacking cells is in the NHEJ DSB-repair pathway, and not HR.

### K-H loss sensitizes cells to chemotherapeutics that induce DSBs

**Figure 3.**
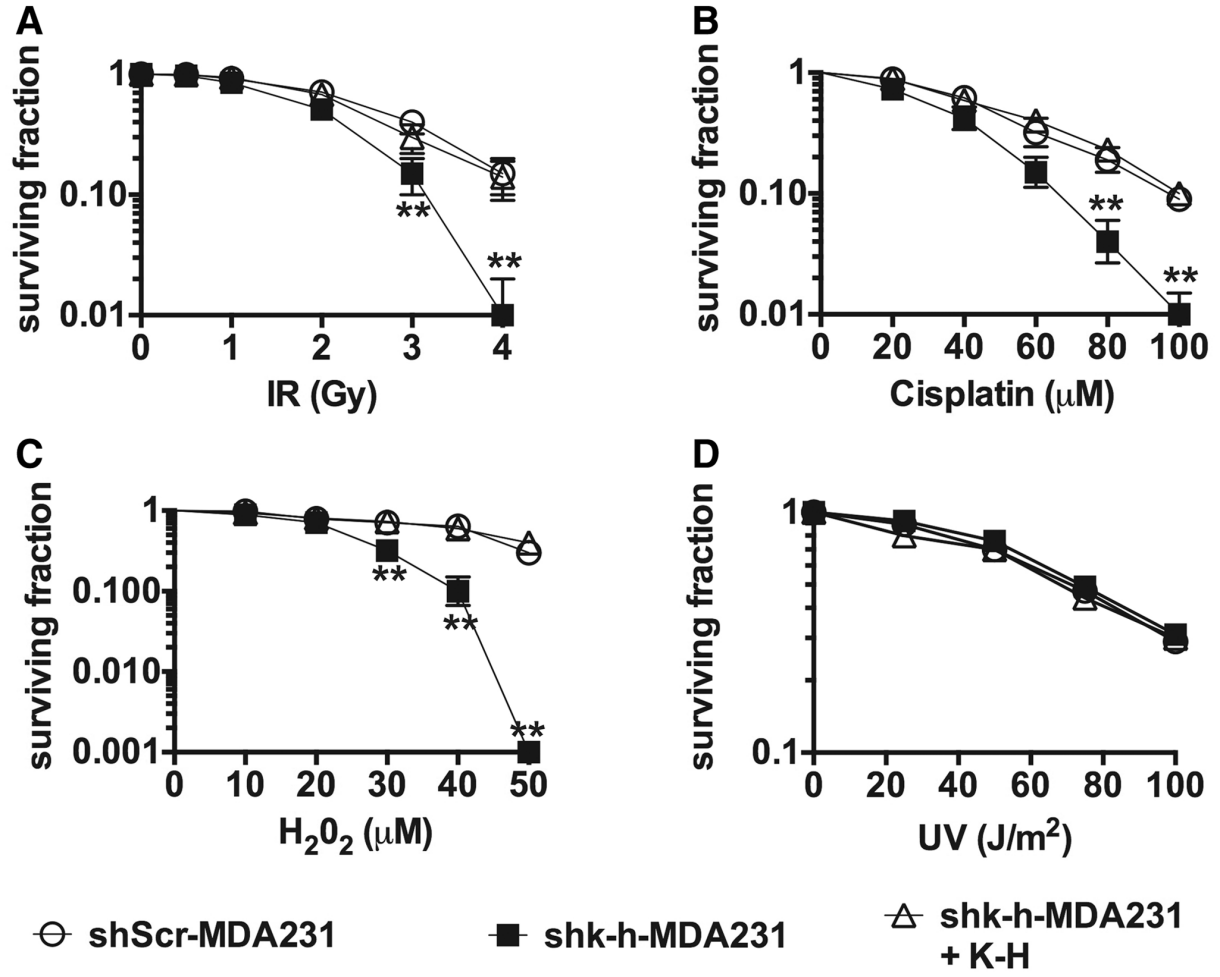
Loss of K-H sensitizes MDA-MB-231 cells to various chemotherapeutic agents. (A–D) shScr-MDA231, shk-h-MDA231 and shk-h-MDA231 + K-H mock untreated or (A) IR, (B) cisplatin, (C) H_2_O_2_, (D) ultraviolent light (UV) exposed cells were monitored for survival by colony formation assay. Colonies were determined as accumulations of at least 50 cells. (**P < 0.01).

Cells deficient in specific DSB-repair pathways typically show unique hypersensitivities to specific cytotoxic agents in long-term colony forming (survival) assays (38,39); for example, NHEJ-deficient Ku70 knockout cells are hypersensitive to agents that specifically create DSBs (40). In general, all cells deficient in K-H (Figure 2A) were hypersensitive to IR treatments compared to mammalian Scr or wild-type (*mk-h*^+/+^) MEF cells (Figure 3A and Supplementary Figure 4A and B). Furthermore, shk-h-MDA231 cells were also hypersensitive to all DSB-inducing agents tested, including cisplatin, H_2_O_2_, Etoposide, Doxorubicin and Topotecan compared to genetically matched shScr-MDA231 cells (Figure 3B and C and Supplementary Figure 4C–E, respectively). In contrast, shk-h-MDA231 cells were not sensitive to ultraviolet (UV) light (Figure 3D). To control for shRNA off-target effects, we reconstituted shk-h-MDA-231 cells with human K-H cDNA and observed that such cells were restored for resistance to IR, cisplatin and H_2_O_2_ treatments to levels comparable to shScr-MDA231 cells (Figure 3A–D). These data demonstrate that K-H is intimately involved in mediating the repair of DSBs and not UV-induced DNA lesions, such as UV-induced thymine dimers and 6–4 photoproducts (41).

### The CID domain of K-H is required for Artemis protein stabilization

**Figure 4.**
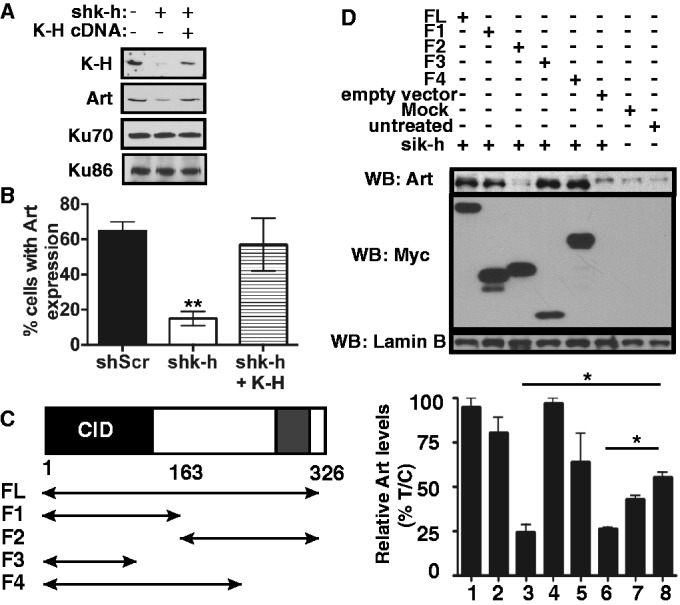
K-H CID domain is essential for stabilization of Artemis. (A) shScr, shk-h and shk-h + K-H were monitored for changes in Ku70, Ku86, Artemis, and K-H expression by western blot. (B) shScr, shk-h and shk-h + K-H cells were assessed for changes in Artemis nuclear location monitored by immunofluorescence. (C) Schematic of K-H deletion mutants tested. (D) K-H domains required for Artemis expression were monitored in 293 T cells transfected with a K-H specific siRNA designed to the 3′-UTR, that were then transfected with wt or mutant K-H plasmid constructs. Cells were harvested and separated by SDS-PAGE and monitored for Artemis, c-Myc and Lamin B by western blot. (*P < 0.05).

As shown above, K-H displays a biochemical association with NHEJ factors, Ku70, Ku86 and Artemis (Figure 1). To determine whether loss of K-H had any effect on the expression levels of NHEJ proteins, we examined their steady state levels in stable shScr compared to shk-h knockdown fibroblasts. Significantly, loss of K-H led to decreased levels of Artemis, but not Ku70 or Ku86. Importantly, Artemis protein levels were restored upon re-expression of human K-H cDNA, monitored by both western blotting and immunofluorescence (IF) (Figure 4A and B). Since Artemis mRNA levels remained unchanged after K-H loss (Supplementary Figure S5A) or K-H restoration, we suspected that loss of Artemis was due to protein instability, and that the K-H protein stabilizes Artemis by protein–protein interaction. To determine regions of K-H required for Artemis stabilization, we generated various deletion mutants of K-H spanning specific domains within the protein (Figure 4C) and overexpressed each of them in 293 T cells deficient in endogenous K-H expression by prior exposure to siRNA specific for the 3′-UTR region of K-H mRNA. K-H and Artemis mRNA and steady state protein levels were confirmed by qRT-PCR (data not shown) and western blotting, respectively. Lamin B protein levels served as loading controls (Figure 4D). Along with full-length K-H, fragments F1, F3 and F4 of K-H caused stabilization of Artemis steady state protein levels (compare lanes 1, 2, 4 and 5 to siRNAk-h + empty vector, lane 6, Figure 4D). In contrast, overexpression of K-H fragment F2 was not able to stabilize steady state levels of Artemis (lane 3) (Figure 4D). These data strongly suggested that the N-terminal CID domain of K-H (minimally represented by the region indicated by K-H fragment F3) was required for Artemis stabilization (Figure 4D).

### Artemis overexpression rescues DNA repair defects in K-H deficient cells

**Figure 5.**
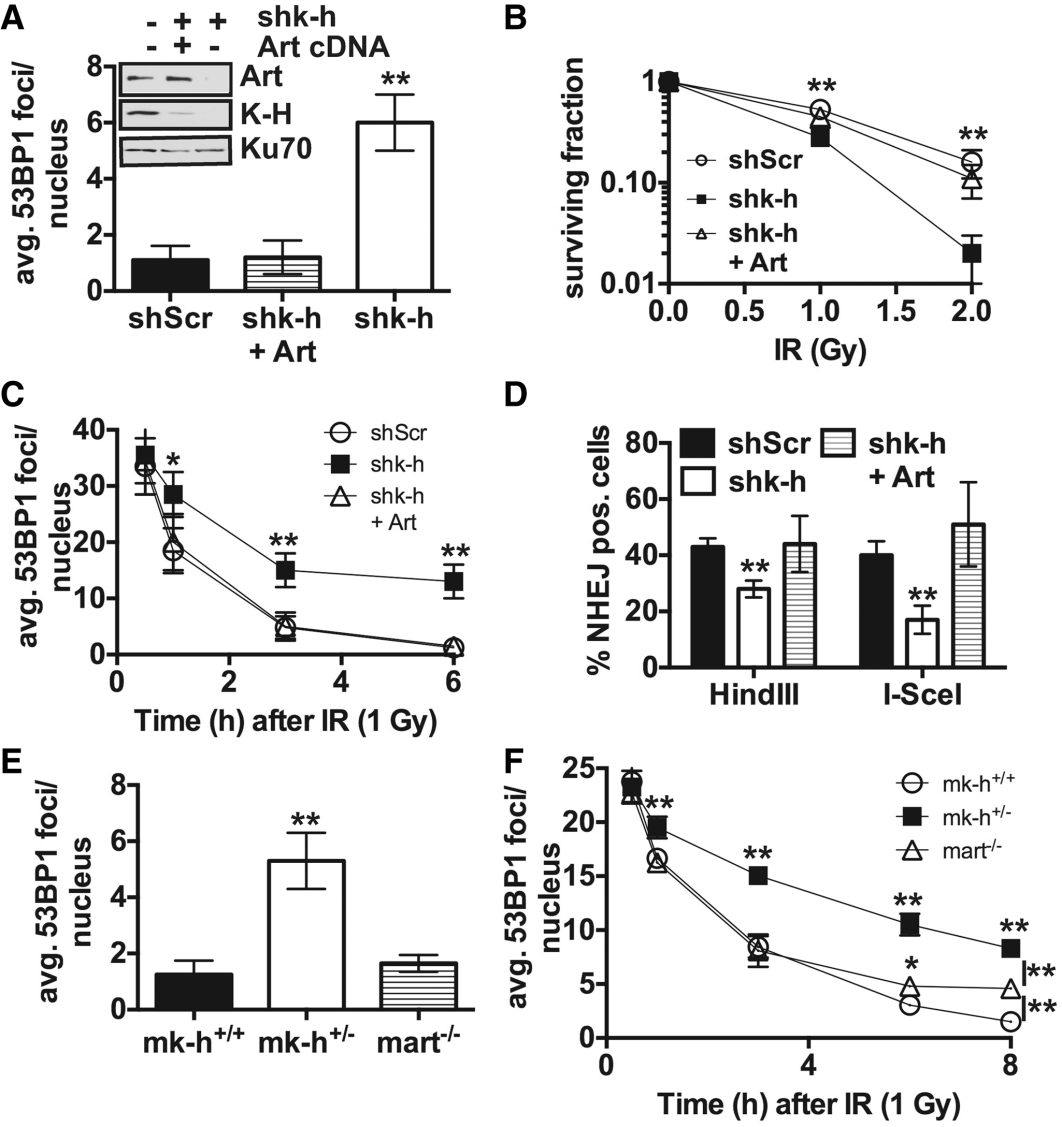
Artemis is required for the DNA-repair capability in K-H deficient cells. (A) Basal levels of the DNA-damage repair biomarker 53BP1 were measured in shScr, shk-h and shk-h cells stably over-expressing Artemis (Art), (shk-h + Art), by immunofluorescence (IF). (B) Sensitivity to IR in shScr, shk-h and shk-h + Art, mock untreated or IR exposed cells was monitored by colony forming assay. (C) Disappearance of 53BP1 foci was measured in shScr, shk-h and shk-h + Art at indicated times after IR, first time point measured is 0.5 h after IR exposure. (D) The ability to perform NHEJ in shScr, shk-h and shk-h + Art was monitored by a plasmid-based NHEJ assay with plasmids digested with either HindIII to study compatible end re-ligation, or I-SceI to study incompatible end re-ligation. (E) Basal levels of 53BP1 foci were measured in wild type (*mk-h*^+/+^), K-H heterozygote (*mk-h*^+/–^) and Artemis deficient (*mart*^–/–^) MEFs by IF. (F) Rate of 53BP1 foci disappearance after IR at times indicated was measured in *mk-h*^+/+^, *mk-h*^+/–^ and *mart*^–/–^ MEFs by IF, first time point is 0.5 h after IR. Cells (300) were visualized for 53BP1 foci. Colonies were determined as ≥50 normal-appearing cells in a 7-day period. Events (10 000) were counted by flow cytometry. (**P < 0.01, *P < 0.05).

Loss of Artemis impairs DNA repair (particularly in the very late phase of DSB repair noted by foci regression), resulting in genomic instability and sensitivity to IR (42). Because K-H loss led to a concomitant loss of Artemis protein expression (Figures 4D and 5A), we examined the effects of Artemis re-expression on the DNA-repair capacity of K-H deficient fibroblasts. We transfected shk-h knockdown fibroblasts with Artemis cDNA (shk-h + Art), which restored Artemis protein expression to levels found in shScr fibroblast cells transfected with the empty vector (Figure 5A and Supplementary Figure 5B). Strikingly, Artemis re-expression in shk-h cells dramatically lowered basal DSB levels detected compared to non-transfected or vector-alone transfected shk-h cells (Figure 5A). Artemis re-expression in shk-h cells also restored resistance to IR and rates of 53BP1 foci regression similar to shScr cells (Figure 5B and C). We then examined whether loss of K-H expression also affected NHEJ capacity in shScr, shk-h and shk-h + Art fibroblasts using a plasmid-based NHEJ assay developed as described (43). These assays employ an mCherry expression reporter plasmid linearized by restriction digestion with HindIII (compatible DNA ends) or I-SceI (incompatible DNA ends) digestion (43). While cells knocked down for K-H expression showed only a slight decrease in the ability to repair compatible ends, there was a more pronounced deficiency in the ability of these cells to repair incompatible DNA ends, similar to results found in *Rtt103Δ* yeast (Figure 5D and Supplementary Figure 1B).

Intriguingly, we also noted that the DNA-repair kinetics, monitored by 53BP1 and γ-H2AX-foci regression, in shk*-*h knockdown cells were significantly and fundamentally different from the DNA-repair kinetic-defects reported in Artemis-deficient cells (42). To explore the mechanistic basis for this difference, we compared the DNA-repair kinetics of wild-type mouse (*mk-h*^+/+^), heterozygote mouse K-H (*mk-h*^+/^^−^) and mouse Artemis^−^^/^^−^ knockout (*mart*^−^^/^^−^) MEFs. *mk-h*^+/^^−^ cells demonstrated a large increase in basal DSB levels compared to *mart*^−^^/^^−^ cells (Figure 5E). In contrast, *mart*^−^^/^^−^ cells exhibited basal DSB levels (averaging 2–4 foci/nucleus) similar to *mk-h*^+/+^ cells. After IR treatment (1 Gy was an equitoxic dose for *mart*^−^^/^^−^ and *mk-h*^+/^^−^ cells), *mk-h*^+/^^−^ cells showed a dramatic impediment in regression of 53BP1 foci/nucleus over time compared to *mart*^−^^/^^−^ or wild-type *mk-h*^+/+^ cells, which are wild-type for Artemis expression (Figure 5F). As previously reported, *mart*^−^^/^^−^ cells showed similar 53BP1 foci/nucleus regression as wild-type *mk-h*^+/+^ cells, except that *mart*^−^^/^^−^ MEFs exhibited a defect in the slow phase of DSB repair, noted at >5 h post-irradiation (Figure 5F). This defect is similar to previously published results using Artemis-deficient human cells (42,44), and strongly suggested that Artemis loss alone was not sufficient to explain the defective DNA-repair phenotype (monitored as foci regression) (Figure 5C and F) observed in *mk-h*^+/^^−^ cells (Figure 5F).

### Loss of K-H leads to R-loop formation

**Figure 6.**
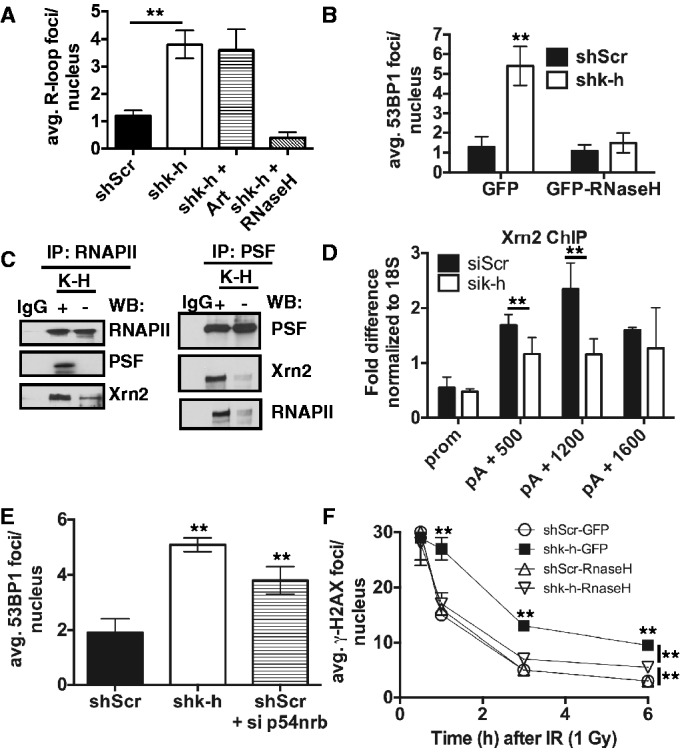
Loss of K-H disrupts transcription termination complexes and leads to R-loop formation. (A) Level of RNA:DNA hybrids (Rloops) in shScr and shk-h cells was measured by immunofluorescence (IF). Basal levels of 53BP1 foci in shScr and shk-h cells transfected with either a GFP-expressing plasmid or a GFP-RNase H-expressing plasmid were measured by IF. (C) Interactions between transcription termination factors PSF and Xrn2 and RNAPII were monitored in shScr and shk-h by co-immunoprecipitation (co-IP) experiments. Co-IPs were performed with RNAPII and PSF specific antibodies and separated by SDS-PAGE. Western blots were then performed with indicated antibodies. (D) ChIP were performed using Xrn2-specific antibody in HeLa cells treated with K-H-specific siRNA or control siRNA at the promoter (prom) and designated points beyond the polyadenylation site. (E) Basal levels of 53BP1-foci formation were measured in shScr, shk-h and shScr cells transfected with p54nrb specific siRNA by IF. (F) Rates of 53BP1 foci disappearance after IR exposure at times indicated were monitored in shScr and shk-h cell transfected with a GFP or and GFP-RNase H expressing plasmid by IF, first time point measure is 0.5 h after IR exposure. (**P < 0.05).

Given previous observations linking K-H to RNAPII transcriptional regulation (28,29), we then examined whether by-products of mis-regulated transcription, in combination with loss of Artemis expression, may explain the qualitative and quantitative differences between the DNA-repair capacities of K-H-deficient and Artemis knockout cells. One such transcriptional by-product is an R loop. Since prolonged R-loop formation leads to increased basal DSBs (45), we monitored R-loop foci/nuclei formation in shk-h and shScr cells by immunofluorescence (IF) using the S9.6 antibody that specifically recognizes RNA:DNA hybrids (31). A considerable increase (∼4-fold) in the number of basal R-loop foci/nucleus was detected in shk-h compared to shScr cells (Figure 6A and Supplementary Figure S6). We also found that Artemis reconstitution had no effect on the level of R loops formed with K-H loss, while R loops were lost when GFP-RNase H was expressed (Figure 6A). Importantly, overexpression of GFP-RNase H, which alleviated R-loop formation similar to prior reports (46,47), significantly decreased basal 53BP1 foci/nuclei in shk-h knockdown fibroblasts to levels detected in shScr cells, with or without RNase H overexpression (Figure 6B): similar effects of RNase H overexpression on R-loop formation and foci representing DSBs were previously reported (46,47). In contrast, transfection of shScr and shk-h cells with a GFP-control plasmid did not alter the increased basal 53BP1 foci/nuclei levels (Figure 6B) noted in shk-h knockdown compared to shScr cells.

To uncouple the contributions of R-loop formation versus Artemis loss and its accompanying specific defect in delayed DSB repair in K-H-deficient cells, we overexpressed GFP or GFP-RNase H in shScr and shk-h cells and exposed these cells to IR (1 Gy). We then monitored γ-H2AX foci-regression rates. While shk-h knockdown cells transfected with GFP alone had similar defective DNA-repair kinetics associated with K-H loss, cells transfected with GFP-RNase H had a distinctly different DNA-repair-kinetic profile (Figure 6F), similar to cells defective in Artemis expression alone (compare repair kinetics in Figure 6F with Artemis-deficient cells in Figure 5F). The DNA-repair kinetics in GFP-RNase H transfected shk-h knockdown cells were similar to GFP-RNase H shScr cells 3 h after IR treatment, but at 6 h a small portion of residual DSBs remained in GFP-RNase H transfected shk-h cells. This response was very similar to that observed with Artemis loss in human cells (42,44). Collectively, our data strongly suggest that R loops directly affect the rate of 53BP1 and γ-H2AX foci regression in early phases of DNA DSB repair in K-H-deficient cells, providing an explanation as to why there is a difference in DNA-repair kinetics in cells with loss of K-H compared to cells deficient in Artemis expression and function.

Yeast Rtt103 interacts with Rat1, the yeast homolog of Xrn2 (27). Rat1 and Xrn2 are 5′–3′ RNA exonucleases required for efficient RNA-transcription termination (48). Accordingly, we noted that the Xrn2–RNAPII–PSF complex observed in shScr fibroblasts was dramatically disrupted in shk-h knockdown cells as monitored by co-immunoprecipitation analyses using antibodies directed toward RNAPII or PSF, a known modulator of Xrn2 (16) (Figure 6C). Kaneko *et al.* (2007) found that Xrn2 localization to the 3′-end of genes is facilitated by PSF (16). In yeast, Rtt103 and Rat1 interact and co-localize to the 3′-end of genes, mediating transcription termination (27). K-H localizes to the 3′-end of the β-actin gene, and loss of K-H leads to increased RNAPII occupancy downstream of the poly A cleavage site of the β-actin gene, suggesting a possible termination defect (28). Loss of Xrn2 also leads to higher RNAPII occupancy at the 3′-end of the β-actin gene (16). We, therefore, assessed whether loss of K-H affected Xrn2 localization along the β-actin gene, contributing to this termination defect. By Chromatin-immunoprecipitation (ChIP) experiments along the β-actin gene we noted that loss of K-H lowered the level of Xrn2 localizing to the 3′-end of the gene (Figure 6D). Therefore, appropriate K-H levels are essential for localizing Xrn2 to transcription-termination regions of DNA. To determine whether loss of other transcription termination factors led to an accumulation of basal DSB formation in our cell model system, we transfected shScr cells with siRNA-targeting p54(nrb), which functions with PSF in termination (16). Indeed, loss of p54(nrb) led to increased basal DSB levels (Figure 6E), similar to previously published results (21).

## DISCUSSION

Consistent with previously published data (30), we demonstrate that *Rtt103Δ* yeast cells can not repair incompatible DSBs. In contrast, these cells were competent at repairing DNA lesions with compatible ends. In human cells, we demonstrate that K-H-deficient cells are also incapable of repairing incompatible DNA ends (Figure 5). Furthermore, we demonstrate that the repair defect in K-H-deficient cells is restored by Artemis re-expression. This suggests that, similar to K-H, the Rtt103 protein might interact with a corresponding yeast protein important for incompatible DSB end processing. While we have no direct evidence for an interaction between these two proteins, yeast defective in Exo1 display a similar DNA-repair defect (34) as noted in *Rtt103Δ* yeast. That is, *Rtt103Δ* and *Exo1Δ* yeast can fully repair plasmid DNA with compatible DNA ends, but are incapable of repairing DSBs with incompatible ends (supplementary Figure 1) (34). Our laboratory is currently investigating whether Rtt103 and Exo1 interact in a manner similar to the association of Artemis with K-H.

K-H-deficient cells demonstrated increased sensitivity to genotoxic stresses that specifically induce DSBs, whereas no hypersensitivity to UV irradiation was noted (Figure 3). Loss of K-H expression greatly suppressed DNA-repair foci regression after a genomic insult with IR, increased genomic instability (Figure 2), and caused a dramatic decrease in the cell’s ability to specifically perform NHEJ (Figure 5). Loss of K-H expression was also accompanied by increased basal DSBs (Figure 2). RNase H overexpression in shk-h cells suppressed basal DSBs (Figure 6), indicating a role for RNA metabolism in the genomic instability observed in these cells.

K-H is known to interact with the CTD of RNAPII (29), and loss of K-H leads to higher RNAPII occupancy at the 3′-end of genes (28). We showed that K-H mediates PSF-Xrn2-RNAPII interactions (Figure 6) and that loss of K-H disrupts these leading to a decrease in Xrn2 recruitment to the 3′-end of genes (Figure 6). These data provide evidence that the RNA PolII termination defect noted in K-H-deficient cells is most likely due to loss of Xrn2 localization.

Importantly, the function of Xrn2 is not limited to the 3′-end of genes. Xrn2 also functions in the premature termination of elongating RNAPII transcription (49,50). Thus, it is not known if the accumulation of persistent and stable R loops is at sites of transcription termination *per se* or at mis-regulated premature termination events. Another interesting possibility is that R loops observed in K-H-deficient cells may be accumulating in the nucleolus. Two interesting studies recently demonstrated that loss of transcriptional regulation of ribosomal DNA leads to R-loop formation and subsequent DSB formation (10,24). Given that Xrn2 plays an integral role in ribosomal RNA trimming (51), it is highly conceivable that the R loops we observe in K-H-deficient cells are accumulating in the nucleolus.

Our data demonstrate that expression of the NHEJ protein, Artemis, was reduced after K-H depletion by transient or stable siRNA knockdown, or through somatic haplo-insufficiency. Since Artemis mRNA levels were not altered in these cells and re-expression of K-H cDNAs containing specific wild-type CIDs restored normal Artemis level, we concluded that K-H is necessary to stabilize Artemis. Interestingly, overexpression of Artemis to over-ride normal degradation processes caused by K-H loss, restored the DNA-repair capabilities and sensitivities to DNA-damaging agents in K-H-deficient cells to levels comparable to wild-type cells. Thus, Artemis expression can repair DSBs created from persistent R-loop formation in K-H deficient cells. This provides insight into the nature of DSB ends created due to Rloops, since Artemis primarily functions on a small subset of DSBs with known substrate specificity, including stem and heterologous loops, flap and gapped substrates (24).

Artemis overexpression resolved R-loop-mediated DSBs, but did not affect persistent R loops (Figure 6) in K-H-deficient cells. We conclude that Artemis acts downstream of RNA:DNA hybrid formation, on DSBs presumably created by DNA or RNA polymerase collisions with R-loop roadblocks. Interestingly, persistent R-loop formation correlates with Histone H3 S10 phosphorylation and chromatin condensation (52), suggesting that the localized chromatin compartment surrounding the Rloop is more heterochromatic than euchromatic. Given that Artemis is required for the repair of DSBs in heterochromatic regions of DNA (44), these findings support our data that DSBs created in response to persistent R-loop formation would require Artemis for repair. Furthermore, RNase H overexpression restored DNA-repair kinetics in shk-h knockdown cells to relatively normal rates similar to those found in Artemis-deficient cells after IR. These data suggest that removal of R loops results only in a defect in the slow phase of DSB repair, similar to that of Artemis-deficient cells. It is known that DSBs in heterochromatic regions of DNA are repaired at a slower rate than DSBs in euchromatin after IR (53). K-H-deficient cells also lose Artemis expression, which is required for efficient slow phase repair of DSBs in heterochromatin (44). R-loop-mediated chromatin condensation would also increase in K-H-deficient cells (52). Couple this with the fact that IR does not adversely affect global transcription rates (54), one would anticipate that cells deficient in K-H expression will have a higher proportion of DSBs with slower DNA-repair kinetics.

Our data provide evidence that K-H exists in multiple protein complexes and with two clear functional properties. K-H associates with several different DNA-repair proteins, such as Ku70, Ku86 and Artemis. In separate complexes, K-H associates with RNAPII and factors that function in transcription termination. While the amino acid sequence of K-H does not appear to contain any obvious functional enzymatic domains, it does contain important protein–protein interaction domains, suggesting that it may function as a molecular matchmaker, or a protein important for functional scaffolding. The coiled-coil region of K-H appears to be dispensable for Artemis stabilization and mediates an interaction with Ku70. Importantly, the CID region of K-H was necessary and sufficient for stabilization of the Artemis protein. CID-domain-containing proteins primarily interact with the CTD of RNAPII (55). Indeed, the CID domain of K-H can associate with the CTD of RNAPII (29). Yet, our data suggest that CID domains may mediate protein–protein interactions independent of RNAPII. The nature of how the CID domain of K-H stabilizes Artemis through protein–protein interactions is unknown and is currently under investigation in our laboratory.

Taken together, our data strongly suggest that loss of K-H copy number and corresponding steady state protein level leads to simultaneous R-loop-derived DSBs and loss of DNA-repair capacity of specific DSBs with complex ends. We theorize that this combination confers a chromosomal ‘mutator’ phenotype on cells and likely contributes to carcinogenesis, since K-H knockdown led to chromatin aberrations. Our data demonstrate that K-H is important in mediating NHEJ, through stabilization of Artemis. In mouse models, Artemis loss was linked to the formation of several different tumor types ranging from pro-B cell lymphomas to sarcomas and malignant gliomas (56,57). Consistent with these mouse data, loss of NHEJ capability and chromosomal translocations are commonly associated with the formation of many different tumor types in humans (58). Interestingly, overexpression of K-H has also been observed in multiple tumor types, such as liver, lung, prostate and colon (28), although current data on K-H deficiency in cancers are extremely limited. In tumors with overexpressed K-H, then, one might expect heightened resistance to genotoxic agents and possibly enhanced cancer progression, particularly if complexes needed in RNA transcription or DNA repair (shown in Figure 1) are functionally altered. Data presented in this article suggest that K-H expression must be critically controlled in the cell for balanced and normal cell growth. Altering its expression may alter its roles as a potential scaffolding protein mediating a balance between transcription termination, genetic instability and Artemis-dependent DSB repair. Loss or gain of expression of K-H may then lead to a chromosomal-level ‘mutator’ phenotype.

## SUPPLEMENTARY DATA

Supplementary Data are available at NAR Online.

Supplementary Data
