# Supplementary material for: Kub5-Hera, the human Rtt103 homolog, plays dual functional roles in transcription termination and DNA repair
Source: Nucleic Acids Res. 2014 Mar 3;42(8):4996–5006. doi: 10.1093/nar/gku160 (PMC4005673; doi:10.1093/nar/gku160)
Supplement: Supplementary Data [file supp_42_8_4996__index.html]

Kub5-Hera, the human Rtt103 homolog, plays dual functional roles in transcription termination and DNA repair — Kub5-Hera, the human Rtt103 homolog, plays dual functional roles in transcription termination and DNA repair — Supplementary Data 

# Kub5-Hera, the human *Rtt103* homolog, plays dual functional roles in transcription termination and DNA repair

## Supplementary Data

files

**Files in this Data Supplement:**

- Supplementary Data - pdf file
