# Supplementary material for: Kub5-Hera, the human Rtt103 homolog, plays dual functional roles in transcription termination and DNA repair
Source: Nucleic Acids Res. 2014 Mar 3;42(8):4996–5006. doi: 10.1093/nar/gku160 (PMC4005673; doi:10.1093/nar/gku160)
Supplement: Supplementary Data [file supp_gku160_nar-03406-d-2013-File002.pdf]

Supplemental Information

Supplemental Figure 1: **K-H is the functional ortholog of yeast Rtt103**. (A) Protein alignment detailing the conservation of Rtt103, mouse K-H, and human K-H proteins were performed using Clustal Omega (59). Previous studies found that K-H and p15RS have high sequence homology and can be found in mammals, birds, fish, and *Arabidopsis (28)*. (B) The ability to perform NHEJ was measured in *hdf1Δ*, *rtt103Δ*, *rtt1103Δ* re-expressing Rtt103 protein, and *rtt103Δ* re-expressing K-H by plasmid based NHEJ with plasmids linearized with BamHI (5' overhang), SacI (3' overhang), BamHI and SacI (in complementary 5'-3' overhang (BamHI+SacI)), and PvuII (blunt ends).

Supplemental Figure 2: **Loss of K-H leads to basal DSBs and delayed 53BP1 foci regression**. (A) K-H antibody specificity was determined by over-expressing K-H and p15RS cDNA in 293T cells. (B) Basal levels of the DNA damage indicators γ-H2AX and 53BP1 were measured in shScr and shk-h cells by immunofluorescence (IF). (C) 53BP1 foci disappearance after IR exposure in shScr and shk-h cells were measured by IF, first time point is 0.5 hours after IR exposure. (D) Neutral comet tail regression after IR exposure in shScr-MDA231, shk-h-MDA231, and K-H overexpressing shk-h-MDA231 cells was measured and quantitated by using Image J (60).

Supplemental Figure 3: **Loss of K-H leads to increased amounts of genomic instability**. (A) Types of genomic aberration in shScr and shk-h cells were

monitored by metaphase spreads: (a) chromatid type damage, (b) chromosome type damage, and (c) di-centric chromosomes.

Supplemental Figure 4: **Loss of K-H leads to hypersensitivity to various genomic insults**. (A-B) Sensitivity to ionizing radiation was measured in (A) shScr, shk-h, (B) *mk-h*$^{+/+}$, and *mk-h*$^{+/-}$ cells by colony forming assay. (C-D) Sensitivity to (C) Etoposide, (D) Doxorubicin, and (E) Topotecan was measured in shScr-MDA231 and shk-h-MDA231 cells by colony forming assay. Colonies were determined as $\geq$50 normal-appearing cells in a 7-day period. (**p<0.01).

Supplemental Figure 5: **Loss of K-H leads to loss of Artemis protein stability**. (A) Levels of Artemis (Art) mRNA were measured in shScr, shk-h, and Art overexpressing shk-h (shk-h + Art) cells by Taqman RT-PCR. (B) Art nuclear localization as visualized in shScr, shk-h, and shk-h + Art cell by immunofluorescence.

Supplemental Figure 6: **Loss of K-H leads to R-loop formation**. R-loop formation was monitored in shScr and shk-h by immunofluorescence using the S9.6 RNA:DNA hybrid antibody.

Supplemental Table 1: **K-H interacts with Ku70**. Yeast Two-Hybrid screen was performed using human full-length Ku70, Lamin B, and an empty expression plasmid as “Bait”. Full-length Human K-H and Human K-H with a L246A mutation was used as “Prey”. The L276A mutation is a negative control for binding based on previous studies that employed a similar mutation (33). Lamin B was used a negative interacting control. Yeast growth on solid media lacking leucine and

tryptophane was used to determine successful transfection of both plasmids. Growth on histidine, adenenine, leucine, and trytophane lacking solid media was used to select successful interacting partners. X-gal positive yeast colonies was were to determine strength of interaction between proteins. Large T antigen was also cloned into pGADT7 “Bait” plasmid and transfected with Lamin B, Ku70, and p53 to test interaction specificities of the yeast two-hybrid assay.

Supplemental Table 2: **Loss of K-H does not elevate all types of genomic instability**. Genomic aberrations were measured in shScr and shk-h fibroblast cells. Tri-radials and Di-centrics were not found to be statistically different by metaphase-spread analysis.

Morales_ Supplemental Fig. 1

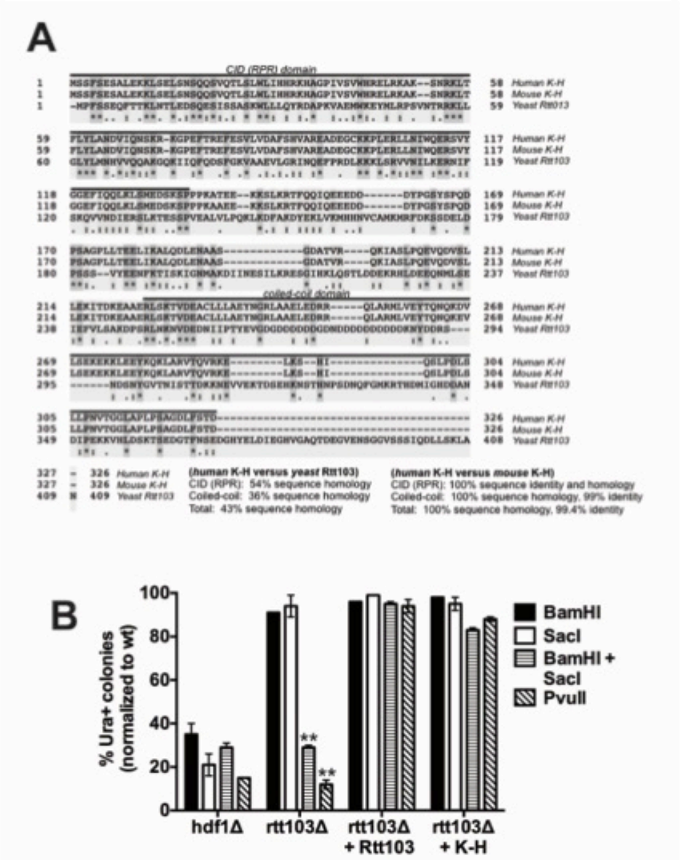

Supplemental Figure 2:

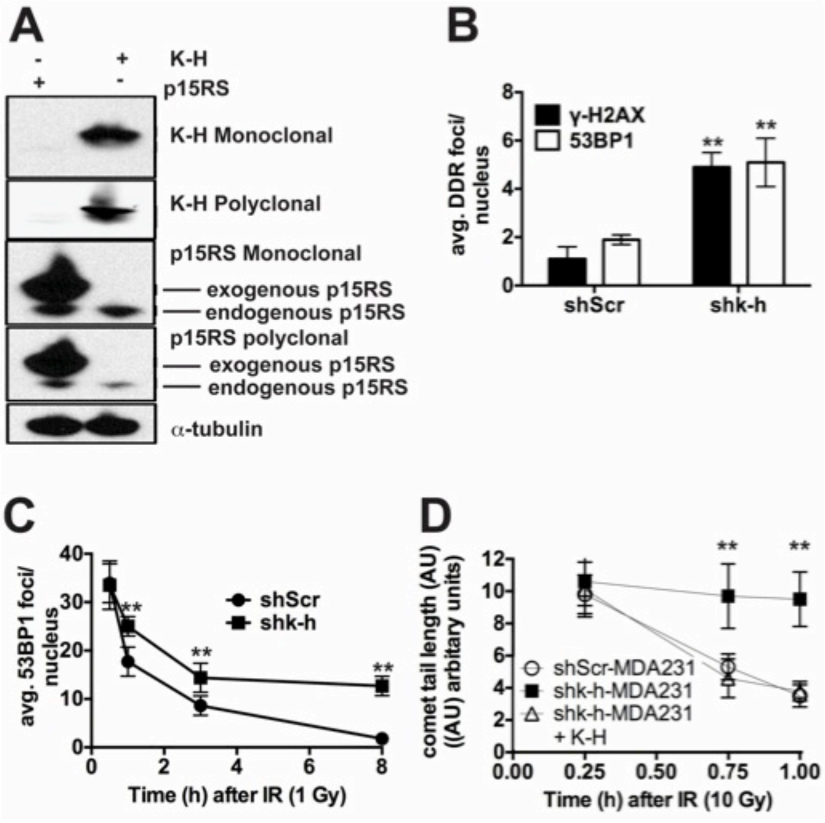

Morales_Supplemental fig. 3

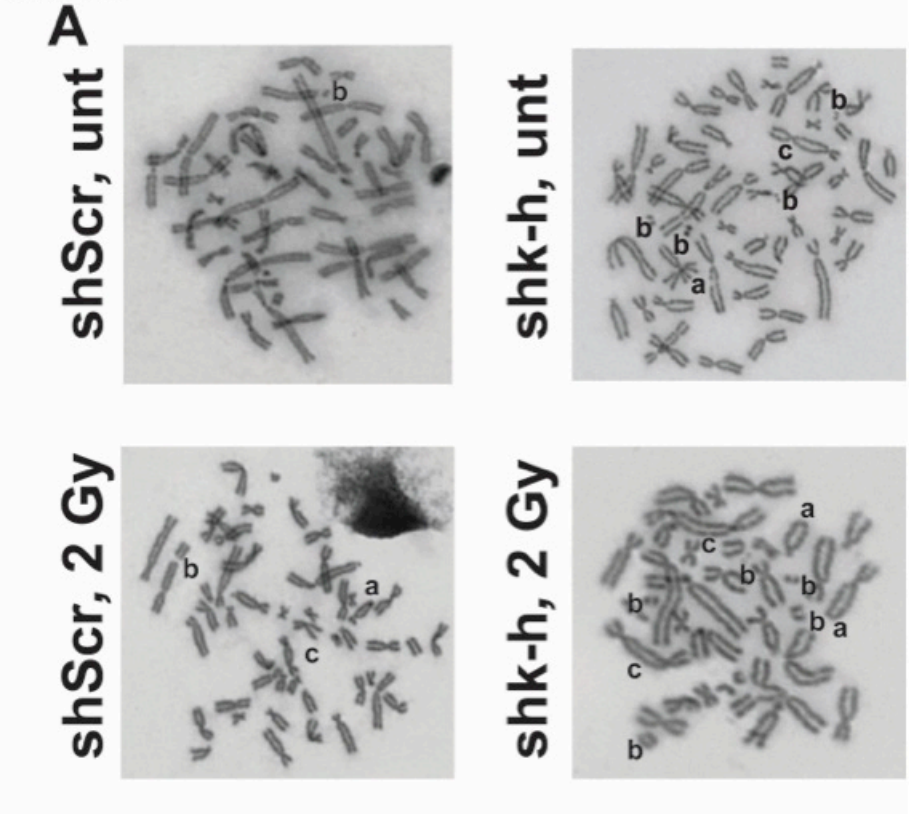

Morales_Supplemental Fig. 4

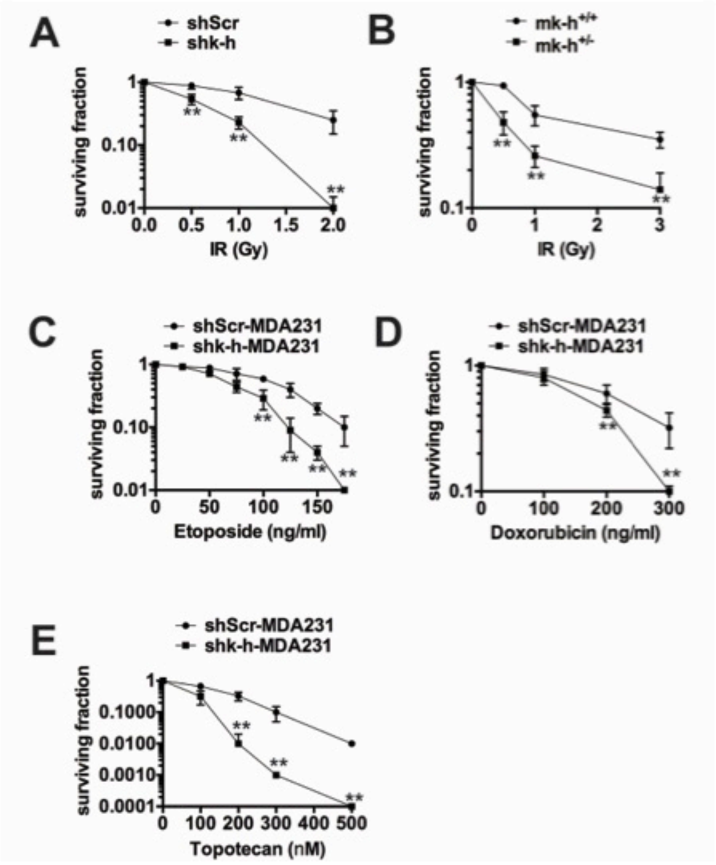

Morales_Supplemental Fig. 5:

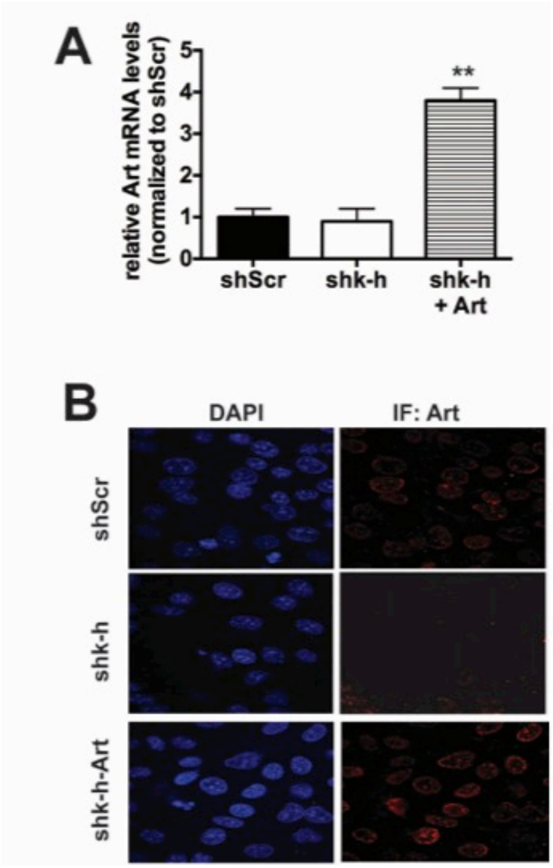

Morales_Supplemental Fig 6:

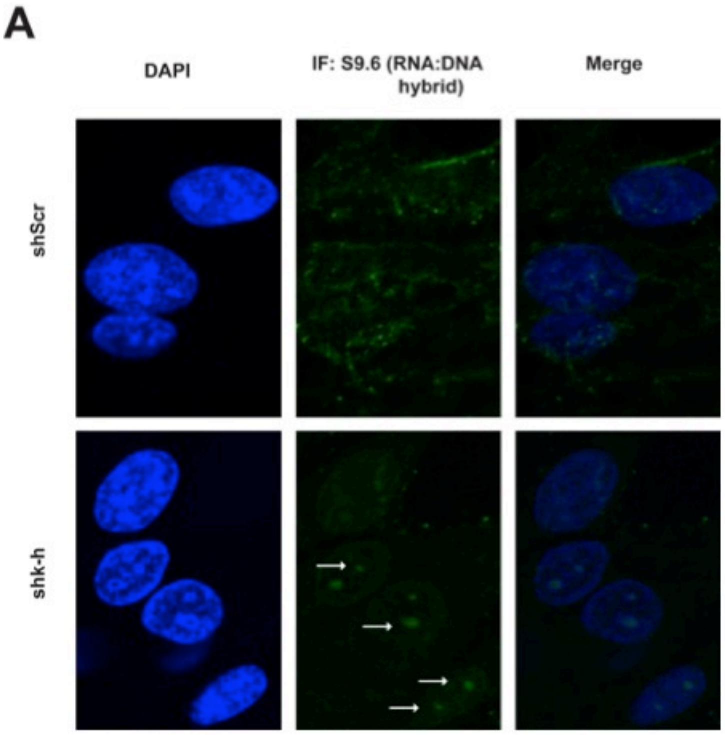

Supplemental Table 1

Interaction		Binding endpoints (growth on specific media)		
Prey	Bait	-Leu-Trp	-His-Ade -Leu-Trp	X-Gal
pGADT7 - K-H	pGBKT7 (empty)	+	-	-
pGADT7 - K-H L276A	pGBKT7 (empty)	+	-	-
pGADT7 - K-H	pGBKT7 - Ku70	+	+	+
pGADT7 - K-H L276A	pGBKT7 - Ku70	+	-	-
pGADT7 - K-H	pGBKT7 - Lamin B	+	-	-
pGADT7 - Large T	pGBKT7 - Lamin B	+	-	-
pGADT7 - Large T	pGBKT7 - Ku70	+	-	-
pGADT7 - Large T	pGBKT7 - p53	+	+	+

Supplemental Table 2:

	aberration/metaphase		
aberration type	shScr	shk-h	p value
Tri-Radials	0.04	0.06	0.65
Di-Centrics	0.59	0.47	0.95
